# Author's reply

**DOI:** 10.4103/0972-2327.48872

**Published:** 2009

**Authors:** Sanjeev V. Thomas

**Affiliations:** Department of Neurology, Sree Chitra Tirunal Institute for Medical Sciences and Technology, Trivandrum – 695 011, India. E-mail: editor@annalsofian.org

Sir,

I am delighted to note that Prof. Dalal[1] has brought out the importance of early intervention to reduce potential disability in stroke survivors. Stroke is a major cause of mortality and morbidity in developing countries as well as more advanced countries. Several studies have confirmed that neurological deficit at onset is a predictor of late sequlae and resultant disability in stroke survivors. The first three to six hours after the onset of stroke is the critical period in which early intervention is most likely to reduce morbidity and final disability. The initial neurological deficit is an indicator of the size of the brain infarction in most situations. Yet, there can be progression of the stroke or added deficits because of increasing cerebral edema. The role of a primary physician to diagnose and institute early treatment for stroke is often underestimated and an efficient stroke center and stroke team is recommended. In India and other countries with resource crunches, it is important that the primary care physicians are appropriately trained to diagnose and institute early treatment for stroke. General care regarding respiration, cardiac care, fluid and metabolic status, control of blood glucose and blood pressure, and prevention of systemic complications are very important. A good review of optimal management of physiological variables during acute stroke was recently published in this journal.[2] Attention to these aspects can be done in any institution with limited facilities yet it forms the first line brain protectant treatment that may reduce the morbidity.
